# Hair Loss and Telogen Effluvium Related to COVID-19: The Potential Implication of Adipose-Derived Mesenchymal Stem Cells and Platelet-Rich Plasma as Regenerative Strategies

**DOI:** 10.3390/ijms23169116

**Published:** 2022-08-14

**Authors:** Pietro Gentile

**Affiliations:** Plastic and Reconstructive Surgery, Department of Surgical Science, “Tor Vergata” University, 00133 Rome, Italy; pietrogentile2004@libero.it; Tel.: +39-3388-5154-79

**Keywords:** COVID-19 and hair loss, COVID-19 and telogen effluvium, SARS-CoV-2 and hair loss, regenerative plastic surgery, PRP in COVID-19, plastic surgery

## Abstract

The diffusion of severe acute respiratory syndrome coronavirus 2 (SARS-CoV-2) inducing coronavirus disease 2019 (COVID-19) has increased the incidence of several dermatological disorders, including hair loss (HL). This article aims to review the literature regarding the incidence of HL and telogen effluvium (TE) in COVID-19 patients and critically appraise the available evidence regarding the role of regenerative strategies like Platelet-Rich Plasma (PRP) and Human Follicle Stem Cells (HFSCs). A literature review regarding the correlation of HL and TE in COVID-19 patients analyzing the biomolecular pathway involved and the role of regenerative strategies was performed using PubMed, MEDLINE, Embase, PreMEDLINE, Scopus, and the Cochrane databases. Observational studies revealed an escalated incidence of pattern HL and TE in COVID-19 patients. Psychological stress, systemic inflammation, and oxidative stress are potential culprits. Proinflammatory cytokines and stress hormones negatively affect the normal metabolism of proteoglycans. Reduced anagenic expression of proteoglycans is a potential mediating mechanism that connects HL to COVID-19. Currently, only one study has been published on PRP against HL in COVID-19 patients. Further controlled trials are required to confirm PRP and HFSCs efficacy in COVID-19 patients.

## 1. Introduction

Hair loss (HL), also known as alopecia or baldness, may be classified in several degrees and kinds. Common types include male- or female-pattern hair loss (MPHL or FPHL), alopecia areata (AA), and a thinning of hair known as telogen effluvium (TE). The cause of MPHL is a combination of genetics and male hormones; the cause of FPHL is yet unclear; the cause of AA is autoimmune, and the cause of TE is typically a physically or psychologically stressful event [1,2,3,4]. Androgenetic alopecia (AGA) is one of the most important and frequent HL causes affecting a mean 80% of white men and 40% of women, determining an MPHL and an FPHL [1,2,3,4], respectively. In AGA, lymphocytes and mast cells have been seen around the miniaturizing follicle detailed in the stem cell-rich lump zone [1,2,3,4]. Miniaturization of the follicles is characterized by a diminishment of the anagen phase, with an improvement in the number of resting hair follicles and telogen, containing microscopic hairs in a hairless scalp [5,6,7]. In HL scalp, hair follicle stem cell numbers stay unaltered, though the number of more actively proliferating progenitor cells particularly diminishes [8]. By 2019, an evolving body of literature associated coronavirus disease 2019 (COVID-19) with primary mucosal, hair, nail, and skin complaints, which may precede the classic symptoms of COVID-19 in some cases. Pruritic erythematous rash and/or patchy exanthemata’s red rash on the trunk appears to be the most common cutaneous finding. Acro ischemic lesions or “COVID toe”, which are micro thrombotic presentations of COVID-19, may occur in both children and adults [9,10]. Hair growth-related disorders have also been an important area of concern during the recent COVID-19 outbreak among both clinicians and the public. A web-based evaluation of public dermatologic interests using Google Trends in Italy and Turkey between April and June of 2020 revealed that hair losses was among the most searched dermatology-related terms in both countries [11]. A simultaneous rise in public apprehension about HL along with the rising number of COVID-19 cases is indicative of a connection. Either the pathogenetic aspects of psychiatric complications of COVID-19 can likely lead to the appearance or aggravation of HL. The present literature review aims to clarify the correlation between HL, TE and COVID-19, analyzing the role of stress and systemic inflammation role, and suggests a potential implication of regenerative strategies for the treatment of hair loss in individuals suffering from COVID-19.

## 2. SARS-CoV-2 Related COVID-19, and Hair Loss

### 2.1. Study Overview

A literature review with the multistep search of the PubMed, MEDLINE, Embase, Pre-MEDLINE, Ebase, Clinicaltrials.gov, Scopus, and Cochrane databases was performed to identify studies published before 1 July 2022, relating to the correlation between HL and severe acute respiratory syndrome coronavirus 2 (SARS-CoV-2), analyzing the biomolecular pathway involved in AGA, the biomolecular impact of SARS-CoV-2 in hair loss, and the potential role of regenerative strategies against SARS-CoV-2, searching without a language or publishing date restriction.

The studies included in this article had to match predetermined criteria according to the PICOS (patients, intervention, comparator, outcomes, and study design) approach. Criteria for inclusion and exclusion are specified as follows: P-Patients (inclusion criteria, age 18–80 years, patients suffering from COVID-19 with TE, or MPHL in stage I–VI controlled by the Norwood–Hamilton classification scale, patients suffering from COVID-19 with TE, or FPHL in stage I–III controlled by the Ludwig classification scale, and patients with TE and/or HL with previous diagnosis of COVID-19 performed by polymerase chain reaction (PCR) or antibody testing. Exclusion criteria included cicatricial alopecia, lichen planopliaris); I-Intervention (inclusion criteria: infiltration or application of autologous platelet-rich plasma, infiltration or application of autologous adipose-derived mesenchymal stem cells, infiltration or application of autologous human follicle stem cells, micro-needling and/or low-level laser/led therapies, minoxidil, finasteride; exclusion criteria: not applied); C-Comparator (inclusion criteria: any type of control, internal, external and different product; exclusion criteria: not applied); O-Outcomes (inclusion criteria: Hair count, hair density, hair thickness and hair color improvement; hair loss reduction; exclusion criteria: Not applied); S-Study design (inclusion criteria: Clinical trial, randomized placebo-controlled trial/randomized, double-blind, placebo- and active-controlled, half-head study/double-blind, placebo-controlled pilot study/blinded, randomized clinical trial, reviews, systematic review expert opinion, comments, letter to editor, case report, preclinical model (animal studies), in vitro, in vivo, ex vivo studies, exclusion criteria: unpublished investigations, conference reports, and lack of raw data.

### 2.2. Historical Context

Since late 2019, the severe acute respiratory syndrome coronavirus 2 (SARS-CoV-2) outbreak has posed a serious threat to human health and wellbeing worldwide. [12]. The human infection COVID-19 is brought on by this strain of the Coronaviridae family. The clinical manifestation of COVID-19 ranges from asymptomatic infection to a multiorgan illness that is potentially fatal. Due to the virus’ affinity for angiotensin-converting enzyme 2 receptors, pulmonary epithelial cells and pneumocytes are the primary target organs of SARS-CoV-2 [13]. Several serious consequences, including pneumonia, acute respiratory distress syndrome, acute liver and/or renal disease, cardiac issues, prothrombotic coagulopathy, and neurological disorders, are reported to be made more likely by COVID-19 [14]. The COVID-19 global pandemic has had a significant psychological impact on the populace. Dermatological problems are another, less well-known, set of complications that this complex disease exposes its victims to [9].

### 2.3. Clinical Studies Analyses Reporting Hair Loss and Telogen Effluvium Increasing in COVID-19 Patients

Clinical investigations performed in Spain [15,16] and India [17] demonstrated higher rates and severity of pattern hair loss (PHL) in patients hospitalized with COVID-19 compared to age-matched, non-infected people. The first preliminary work was a descriptive study on 41 Caucasian males admitted to hospitals in Spain with a diagnosis of bilateral SARS-CoV-2 pneumonia (mean age = 58 years). Seventy-one percent of the patients were diagnosed with significant MPHL, of which 39% had a severe involvement [15]. A follow-up, multicenter study on 175 confirmed COVID-19 patients verified those preliminary findings and reported that 79% (95% CI: 70–85%) of men and 42% (95% CI: 29–55%) of women had significant PHL. The projected prevalence rates among age- and race-matched populations are in stark contrast to these figures. In a comparable white population, it is predicted that MPHL will be prevalent in the range of 31–53% and FPHL will be prevalent to a maximum of 38% [16,18]. Therefore, the information currently available indicates that hospitalized COVID-19 patients have a much greater prevalence and severity of PHL. Notably, patients with more advanced HL had worse clinical results (use of ventilators and deaths). The phenomenon of severe baldness in COVID-19 patients with a higher probability of negative outcomes was named the “Gabrin sign” by some researchers [19]. It is unquestionably necessary to conduct bigger, controlled surveys in both inpatient and outpatient settings to determine whether or not this association is caused by a causal relationship. Diffuse alopecia appears to be a significant COVID-19 aftereffect, in addition to having a favorable connection with the SARS-CoV-2 infection. In order to investigate the prevalence and predictors of COVID-19 clinical sequelae, a sizable longitudinal study with 538 COVID-19 survivors and 184 controls was conducted in Wuhan, China [19]. Alopecia was one of the most common complaints among COVID-19 convalescent patients three to four months after discharge, with women reporting it more frequently. Almost half of the female participants began losing their hair after contracting SARS-CoV-2, in contrast to the control group, which had no cases. Seventy-three percent of affected people initially saw their alopecia after being discharged, while 27% of affected cases had it while they were hospitalized [19]. Due to the timing of symptoms, at least a proportion of the cases with newly-onset alopecia in this study are suspected to have premature or exacerbated FPHL. An explanation of the observed relationship between PHL and COVID-19, a key role for systemic inflammation as a common underlying pathology, is conceivable. This important factor may as well justify higher grades of hair loss in patients with severe COVID-19 as reported by Wambier et al. [17] hypoxemia leading to skin ischemia is another potential pathogenetic factor that connects lung damage secondary to SARS-CoV-2 infection with hair growth impairment. 

The analyzed studies have been summarized in Table 1.

Regarding the correlation between TE and COVID-19, a study of Olds et al. [20] identified 10 patients with TE attributed to COVID-19 infection and described their presentations as a case series. About 80% of these patients were treated with antibiotics, systemic corticosteroids, and/or hydroxychloroquine for their COVID-19 infection, and 70% were hospitalized. The presentations of these patients suggested that COVID-19 infection may be a significant trigger of TE. According to Olds et al. [20], TE caused by hydroxychloroquine, azithromycin or other medications cannot be ruled out, and the global pandemic itself is a source of psychosocial stress. Another study of Sharquie, K.E et al. [21], described the possible effects of COVID-19 on the hair growth cycle and the relationship between COVID-19 and acute TE. In this observational cross-sectional study, conducted from September 2020 to March 2021, 39 patients with post COVID-19 HL are confirmed by PCR or antibody testing. A hair pulls test was carried out to confirm the diagnosis and severity of TE. All experienced excessive hair loss within 2–3 months after infection. Pull tests were strongly positive (>10–50% with a mean of 35% of pulled hair away from scalp). The correlation between COVID-19 and TE has been demonstrated also by a multicentric study conducted by Moreno-Arrones et al. [22] which enrolled 214 patients from March to August 2020 with acute TE that had a prior SARS-CoV-2 infection. The mean number of days since SARS-CoV-2 diagnosis and significant hair shedding was 57.1 days (SD of 18.3). A history of fever was associated (*p* = 0.04) with increased hair shedding (Sinclair score of 5 or 6) while the use of heparinoids was not associated with severity.

Interestingly, Shome et al. [23] reported that 20 adult patients (all women) showed TE episodes a few weeks after recovery from COVID-19 infection, continuing for more than six months. 

Hussain et al. [24] performed a systematic review involving 465 patients diagnosed with acute TE. The mean age was 44 years, and 67.5% were women. The most common trichoscopy findings were a decrease in hair density and empty follicles. The average duration from the onset of COVID-19 symptoms to the appearance of acute TE was 74 days, earlier than classic acute TE.

The most recent observational cross-sectional study included 198 patients and was published in May 2022 by Seyfi S. et al. [25], and confirmed that TE is one of the consequences of the COVID-19 pandemic. The authors affirmed that COVID-19triggers TE via the necessary medications’ treatment and stress situations.

Monari et al. [26], in a cross-sectional study which enrolled 96 patients with a diagnosis of SARS-CoV-2 pneumonia, identified TE in 31.3% of patients, with a significant difference in sex (females 73%, males 26.7%). The average time detected from the onset of the first symptoms to TE was 68.43 days. Overall, there were no significant associations between TE and COVID-19-related features (length of hospitalization, virologic positivity, or duration of fever), treatment characteristics, or laboratory findings. The authors concluded that post-infection acute TE occurs in a significant number of COVID-19 patients and the burden of this condition may impair the quality of life, with a significant impact on individuals.

The analyzed studies have been summarized in Table 2.

### 2.4. The Role of Systemic Inflammation, Oxidative Stress, and Ischemia in COVID-19 Related Hair Loss 

Although MPHL and FPHL are traditionally categorized as non-inflammatory types of hair loss, it is becoming progressively evident that immune-driven pathways and inflammation are inseparable causal elements [27]. Recent attention to the significance of mechanisms beyond androgens in the pathogenesis of MPHL and FPHL has given rise to a ‘paradigm shift’. This modern perspective has substantial clinical implications on the choice of treatment depending on the possibility and extent of inflammation/oxidative stress in everyone [28,29]. Here it is proposed that immune-driven reactions are among the main etiological factors of COVID-19-related diffuse hair loss. There is direct histological evidence for the involvement of inflammation in PHL. Examination of biopsies from transitional scalp areas of patients uncovered extensive infiltration of mononuclear cells and actively degranulating mast cells within follicular sheaths. Fibroblastic activation in alopecic areas resulted in the deposition of collagen and the replacement of follicular technogenic elements by fibrotic sheath residua (fibrous tracts). In addition, soluble materials and cytokines secreted by infiltrating immune cells may also exert deleterious effects on the cyclic activation of papillary cells and stem cell populations [30]. Overproduction of proinflammatory cytokines, including interleukin 1 (IL-1) and tumor necrosis factor α (TNF-α), induces premature catagen, triggers oxidative stress, and promotes apoptosis in hair cells. Keratinocytes are shown to respond to chemical stress within minutes by releasing such factors as IL-1, reactive oxygen species (ROS), prostaglandins, and histamine. These diffusible factors potently inhibit hair growth and survival [31]. Oxidative stress in follicular microenvironments, which is a known contributor to PHL [32], can be triggered by several of the main etiologies of alopecia, e.g., drugs, stress, age, and exposure to microbial antigens. The role of oxidative stress and related ischemia in hair loss has been described in ex vivo and in vivo experiments by Kato et al. [33], demonstrating significant reductions in hair growth rate, hair shaft size, and pigmentation in anagen hairs during situations of blood flow reduction.

SARS-CoV-2 is a cytopathic virus capable of causing high levels of virus-linked pyroptosis and vascular leakage in involved tissues [13]. Thus, local and systemic inflammation are pivotal pathogenetic sources of tissue damage and systemic complications in acute and convalescent COVID-19 patients. Cell invasion and disseminated pyroptosis trigger a strong cytokine response boosting the plasma levels of major proinflammatory cytokines: IL-1β, IL-6, IL-2, IL-17, interferon γ (IFN-γ), monocyte chemoattractant protein 1 (MCP-1), IP-10, and many more. It is sensible to hypothesize that such an abrupt surge in the circulating level of multiple catagenic cytokines in COVID-19 patients exposes follicular cells to strong inhibitory and disruptive influences [13,15,16,17,18,32]. In response, the hair growth cycle becomes disrupted and the gradual process of PHL greatly accelerated [13,15,16,17,18,32]. This mechanism explains the appearance of exacerbated hair loss shortly after being infected with SARS-CoV-2, as described earlier. Accordingly, inflammation and oxidative stress appear to play determining roles in COVID-19-related hair loss and need to be taken into consideration in our clinical approach [13,15,16,17,18,32]. Thus, the role of inflammation, as well as the lack of a safe conventional treatment to address this pathology, has contributed to the common undertreatment and dissatisfaction of patients. 

**Figure 1:**
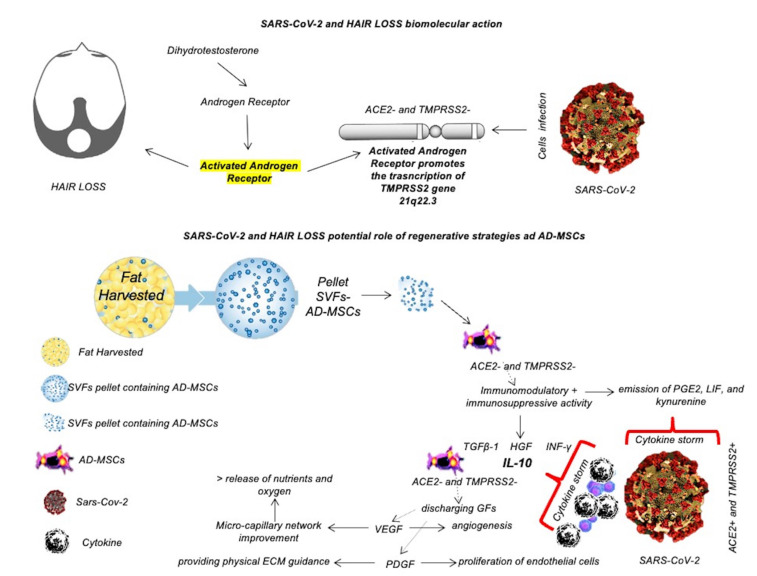
Biomolecular pathway implicated in SARS-CoV-2 and hair loss in comparison with the potential biomolecular actions of AD-MSCs against SARS-CoV-2.

A graphic illustration of the biomolecular pathway implicated in SARS-CoV-2 infection has been reported in Figure 1. 

### 2.5. The Correlation between Inflammation-Related Covid-19 Related and Telogen Effluvium

TE is typically a physically or psychologically stressful event [1,2,3,4] and is the most common diffuse type of hair shedding because of the premature termination of anagen and hair follicle entry into catagen. As reported, SARS-CoV-2 triggers a strong cytokine storm, increasing the levels of IL-1β, IL-6, IL-2, IL-17, IFN-γ, MCP-1, IP-10. It is sensible to conjecture that such an abrupt surge in the circulating level of multiple catagenic cytokines in COVID-19 patients exposes follicle cells to strong inhibitory influences [13,15,16,17,18,32], promoting the hair follicles enter quiescence, outcoming to acute TE episodes [34]. In fact, the inflammatory cytokines, including IL-6, TNFα, IL-1β, and IFN-γ, develop the catagen cycle in experimental studies has been demonstrated [35]. Furthermore, anticoagulant proteins are decreased due to the anticoagulation cascade in response to COVID-19, leading to microthrombi formation and obstructing hair follicle blood supply. These factors could be considered precipitating factors of TE after COVID-19 infection [36,37].

**Figure 2:**
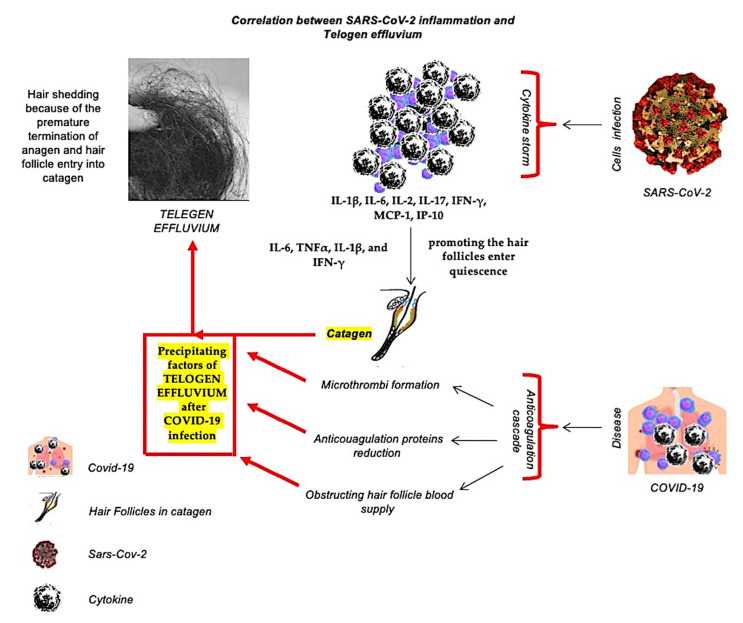
A graphic illustration of the correlation between the inflammation produced by SARS-CoV-2 cell invasion and Covid-19 disease with telogen effluvium.

A graphic illustration of the correlation between inflammation related Covid-19 and Telogen effluvium has been reported in Figure 2. 

## 3. The COVID-19 Pandemic Provoked Stress-Induced Hair Loss

Since its start, COVID-19 has had a widespread, well-known effect on society that has extended far beyond the boundaries of the infection itself: anxiety and tension. The World Health Organization (WHO) has noted a sharp increase in the prevalence of stress and anxiety worldwide. The psychological impact of COVID-19 has been significantly determined by several crucial elements, including forced life adjustments, economic insecurity, and the fear of the unknown. It is anticipated that the self-isolation and quarantine measures that interfere with people’s daily activities will raise the incidence of sadness, anxiety, substance addiction (including alcohol and drugs), and suicide [38]. During a global pandemic, there is a higher risk of psychiatric difficulties for all affected individuals, healthcare professionals, and the public. 

Several “stress-sensitive” skin conditions, such as acute and chronic telogen effluvium (TE), have a documented origin in psychological stress [39,40]. The nature and timing of the stress have an impact on its physio-pathological effects. It has been determined that there are three different kinds of psychological stress: (1) positive stress, which is a moderate, brief, and unavoidable component of daily life; (2) tolerable stress, which is more intense but occurs infrequently and gives the brain time to recover; and (3) toxic stress, which is significant in magnitude and results in the prolonged activation of systemic stress responses, including the sympathetic adrenomedullary system. Chronically increasing the levels of cortisol and catecholamines in the blood can cause a variety of illnesses, including anxiety, hypertension, depression, chronic pain, autoimmune diseases, and cancer [41].

Stress has been demonstrated to dramatically increase premature catagen and intrafollicular apoptosis in hair follicles on the scalp in in-vivo studies. Due to numerous anagen terminating signals induced by the virus, TE associated with COVID-19 is likely to be of an “immediate anagen release” kind with substantial loss of club hairs [42]. By influencing follicular stem cells and mis-regulating the metabolism of follicular proteoglycans, elevated cortisol, and catecholamine levels are said to change the hair growth cycle [43,44]. Perifollicular inflammation manifested by activated macrophage clustering and mast cell degranulation was observed in subjects experiencing psychological stress [40]. Additionally, people with severe COVID-19 frequently get a variety of pharmacologic treatments, such as anticoagulants, which can individually cause TE. Therefore, TE is at least partially to blame for the increased rate of hair loss in COVID-19 survivors that was detected in >27% of the 1100 survivors in a net-based survey [45] and reported by Xiong et al. [19]. Although in-person referrals to dermatological clinics decreased during the COVID-19 pandemic, a more focused investigation found that the incidence of TE was higher than it had been prior to the pandemic [46]. This study is in keeping with the personal accounts shared online since the emergence of COVID-19 by several healthy people, COVID-19 survivors, and dermatologists from around the world [45,47,48].

## 4. Regenerative Therapies in COVID-19 Related Hair Loss

As reported, hypoxia in COVID-19 patients determines detrimental effects on hair growth and hair cycling, which could justify the clinical application of Platelet-Rich Plasma (PRP) and alternative regenerative therapies with protective effects against ischemic injury, including Human Follicle Stem Cells (HFSCs) and/or Micro-needling and/or Low-Level Light Therapy (LLLT). All of these therapies aim to improve scalp angiogenesis. [49,50,51,52,53]. On the other hand, current treatments for MPHL and FPHL approved by the United States (US) Federal Drug Administration (FDA) are oral Finasteride^®^ and topical Minoxidil^®^ in various forms, including solution and foam [52].

Instead, TE occurs because of an induced imbalance in the dynamics of the hair growth cycle secondary to several triggering factors. These triggers are assumed to shift the equilibrium between anagenic and catagenic pathways in follicular cells and thereby initiate premature catagen. In individuals who suffer from COVID-19, the intense psychological pressure and massive inflammatory response are plausible triggers for acute/chronic TE [19]. Inflammation per se can act as an independent as well as a mediating etiology. In vivo research demonstrated that specific blockage of the master regulator of inflammation, nuclear factor κB, can largely neutralize the effect of stress on hair follicles [40]. This interesting finding suggests that agents with anti-inflammatory properties have a worthy potential to be used in the clinical management of TE associated with the COVID-19 pandemic. 

In the absence of a conventional and standardized therapeutic option, PRP and all the regenerative strategies aiming to improve the angiogenesis and to reduce the inflammation could be deemed useful and indicated in treating HL and acute/chronic TE triggered by COVID-19 or its psychological consequences. This potential stems from two properties of PRP as described earlier: first, the promotion of angiogenesis performed by the PRP through the growth factors (GFs) release (especially VEGF), and second, its in-vivo anti-inflammatory properties [50]. Mitigating the impact of inflammatory cytokines and stress hormones on the activity of follicular cells can prevent premature catagen initiation. On the other hand, enhancing the angiogenesis (through VEGF) and promoting the survival of dermal papilla cells during the hair cycle via the Bcl-2 protein’s activation (antiapoptotic regulator) and Akt signaling aim to promote anagen [50]. The stimulation of Wnt signaling in dermal papilla cells is considered a key factor in promoting hair growth. Mesenchymal stem cell-derived signaling and GFs obtained by PRP influence hair growth through cellular proliferation to prolong the anagen phase (FGF-7), induce cell growth (ERK activation), stimulate hair follicle development (β-catenin), and suppress apoptotic cues (Bcl-2 release and Akt activation) [50]. This dual therapeutic activity has the potential to prevent the progression of HL and help accelerate hair regrowth in COVID-19 survivors and non-infected individuals struggling with stress-induced TE. Currently, only one paper has been published on the use of PRP (by Talei et al.) [53] in a female who suffered non-scarring alopecia following COVID-19 hospitalization and an intensive care stay where she lost a large percentage of her hair, with encouraging results. Another factor that deserves consideration is the role of increased cortisol under the conditions of COVID-19 [54]. High cortisol levels relate to the increased degradation of matrix proteoglycans and is, therefore, considered a disruptor of the hair growth cycle [43]. In summary, both biological and psychological factors play distinct roles in driving TE in COVID-19 survivors. Mesenchymal Stem Cells (MSCs), and Adipose-Derived Mesenchymal Stem Cells (AD-MSCs) have been used for many years as regenerative therapies in several fields, including hair loss [49,50,51,52]. In the last two years (2020–21), several studies on their use in patients affected by COVID-19 reported improved respiratory activity after the intravenous administration of MSCs, suggesting their role as an anti-viral therapy [55,56]. Severe COVID-19 patients usually progress to acute respiratory distress syndrome, sepsis, metabolic acidosis that is difficult to correct, coagulation dysfunction, multiple organ failure, and even death in a short period after onset [55,56]. Currently, there is still a lack of clinically effective drugs for such patients. The high secretory activity, the immune-modulatory effect, and the homing ability make MSCs and AD-MSCs both a potential tool for the anti-viral drug-delivery in the virus microenvironment and potential cellular therapy [57,58]. AD-MSCs as the most important exponent of MSCs are expected to reduce the risk of complications and death in patients due to their strong anti-inflammatory and immune-modulatory capabilities, which can improve the microenvironment, promote neovascularization, and enhance tissue repair capabilities [59,60]. For these reasons, the role of regenerative strategies through MSCs, AD-MSCs, and adipocyte-secreted exosomal microRNAs as a potential antiviral therapy needs to be deeply evaluated, comparing the results found with current research progress on drugs in COVID-19 disease. A graphic illustration of the potential biomolecular action of AD-MSCs against SARS-CoV-2 has been reported in Figure 1. 

## 5. Clinical Studies on PRP, HFSCs, and/or AD-MSCs Effects in AGA and Correlations between AGA and SARS-CoV-2

Several papers have been published on the use of autologous PRP [61,62] in patients suffering from androgenic alopecia (AGA), with interesting and encouraging results highlighting the effectiveness of this kind of procedure in patients with low and/or a moderate degree of AGA classified according to the Norwood-Hamilton scale [61,62]. Additionally, new regenerative strategies have been introduced since 2017 in AGA treatment using an autologous suspension of HFSCs obtained from scalp biopsies of the patients, showing similar results to PRP [49,51,63]. 

AGA is a type of androgen-dependent hair loss characterized by miniaturization, progressive microencapsulation of hair follicles, and continuous shortening of the hair follicle growth period [64,65]. It is currently the most common type of hair loss affecting the patient’s appearance, mental health, and social mood. 

Dihydrotestosterone, obtained by the conversion of testosterone through the 5-alpha-reductase function, promotes the activation of androgen receptors (ARs), causing hair loss. Activated ARs promote the transcription of transmembrane protease serine 2 (TMPRSS2) gene 21q22.3, as graphically reported in Figure 1. TMPRSS2 is the spike protein for SARS-CoV-2 [66], and its transcription promoted by ARs strongly activated in AGA could favor the entrance of SARS-CoV-2, leading to the worsening of hair loss until the TE. This concept is the rationale of the present literature review. 

In fact, SARS-CoV-2 cell entry and subsequent infectivity are mediated by androgens and the androgen receptors through the regulation of TMPRSS2, as also postulated by Cadegiani et al. [67]. In this way, AGA predisposes males to COVID-19 disease, while the use of 5-alpha-reductase inhibitors (5ARis) and androgen receptor antagonists reduce COVID-19 disease severity [67].

In the interesting study of Cadegiani et al. [67], the authors aimed to determine the potential benefit of dutasteride, a commonly used broad and potent 5ARi, as a treatment for COVID-19. In detail, all the subjects with a positive reverse transcription-polymerase chain reaction (RT-PCR) test taken within 24 h of recruitment presented with mild to moderate symptoms. Subjects were given either dutasteride 0.5 mg/day or a placebo for 30 days or until full COVID-19 remission. All subjects received standard therapy with nitazoxanide 500 mg twice a day for six days and azithromycin 500 mg/day for five days. The main outcome(s) and measure(s) were as follows: time to remission, oxygen saturation (%), positivity rates of RT-PCR-SARS-CoV-2, and biochemical analysis [ultrasensitive C-reactive protein (usCRP), D-dimer, lactate, lactate dehydrogenase (LDH), erythrocyte sedimentation rate (ESR), ultrasensitive troponin, and ferritin]. Results Subjects taking dutasteride (n = 43) demonstrated reduced fatigue, anosmia, and overall disease duration compared to subjects taking a placebo (n = 44) (*p* < 0.0001 for all). Compared to the placebo group, on Day seven, subjects taking dutasteride had a higher virologic remission rate (64.3% versus 11.8%; *p* = 0.0094), higher clinical recovery rate (84.7% versus 57.5%; *p* = 0.03), higher mean [standard deviation: SD], oxygen saturation (97.0% [1.4%] versus 95.7% [2.0%]; *p* = 0.02), lower median [Interquartile range: IQR] usCRP (0.34 mg/L [0.23 mg/L–0.66 mg/L] versus 1.47 mg/L [0.70 mg/L–3.37 mg/L]; *p* < 0.0001), lower median [IQR] lactate (2.01 mmol/L [1.12 mmol/L–2.43 mmol/L] versus 2.66 mmol/L [2.05 mmol/L–3.55 mmol/L]; *p* = 0.0049), lower median [IQR] ESR (5.0 mm/1 h [3.0 mm/1 h–11.0 mm/1 h] versus 14.0 mm/1 h [7.25 mm/1 h–18.5 mm/1 h]; *p* = 0.0007), lower median [IQR] LDH (165 U/L [144 U/L–198 U/L] versus 210 U/L [179 U/L–249 U/L]; *p* = 0.0013) and lower median [IQR] troponin levels (0.005 ng/mL [0.003 ng/mL–0.009 ng/mL] versus 0.007 ng/mL [0.006 ng/mL-0.010 ng/mL]; *p* = 0.048). The findings from this study suggest that in males with mild COVID-19 symptoms undergoing early therapy with nitazoxanide and azithromycin, treatment with dutasteride reduces viral shedding and inflammatory markers compared to males treated with placebo [65,67].

A significant number of studies have indicated that the dysregulation of lncRNAs is strongly correlated with the onset and development of AGA [68,69]. HFSC aging characterized by the loss of stemness and by epidermal commitment leads to the progressive miniaturization of hair follicles, representing the critical mechanism of AGA [70]. To date, accumulating evidence has indicated that activating HFSC is the new focus of treatment of AGA. Zhang et al. [71] found that VEGF significantly reduced 5α-dihydrotestosterone-induced HFSC apoptosis by inhibiting the PI3K-Akt pathway, thereby delaying the progression of AGA. 

Angiogenesis involves the stimulation of endothelial cells by pro-angiogenic signals, such as VEGF, that is prevalently released by PRP. Promoting angiogenesis and protecting the cells from ischemia are regarded as important mechanisms of action in treating hair loss. While the PRP technique may represent a valid regenerative strategy to improve hair re-growth thanks to its capacity to release several GFs, promoting the survival of dermal papilla cells (DPCs) during the hair cycle via the Bcl-2 protein’s activation (antiapoptotic regulator) and Akt signaling, as already reported, the clinical use of HFSCs to enhance hair re-growth has not been satisfactorily considered. In recent papers [49,51,63,72], the authors cited the amount of CD44+ cells (hair follicle-determined mesenchymal SCs) from the dermal papilla, and the level of CD200+ cells (hair follicle epithelial-SCs) from the bulge, obtained using the customized centrifugation of several punch biopsies [49,51,63,72]. The authors reported the microscopic evaluation of punch biopsy samples, performed using cytospin, immunocytochemistry, and the histological examination achieved by hematoxylin and eosin staining and clinical appraisal, where they discussed improvements to the current systems available for the recovery and regeneration of hair follicles. The authors emphasized permitting neo-genesis of HFs in adult individuals using isolated cells and biotechnologies.

On the other hand, emerging evidence points to the significance of many lncRNA participating in a variety of biological functions by interacting with miRNA and regulating its target genes [71,73]. 

Previous studies have shown that the concentration of immune-inflammatory cells in the bulge area of the hair follicle leads to disorders of the hair follicle microenvironment, thus impairing the normal function of HFSC and resulting in alopecia [74]. Vascularization is closely related to hair growth [50]. On the one hand, the vascular system plays a vital role in maintaining the HFSC microenvironment; on the other hand, stimulating angiogenesis helps to increase the blood supply of DPCs and to promote hair growth. 

TGF-β1 and Wnt signaling pathways are the most crucial pathways that maintain a quiescent niche and regulate the proliferation and differentiation of HFSC [75,76]. Previous studies have reported that TGF-β1 enables hair follicles to enter telogen from anagen in advance, while the transition from the anagen to the telogen of the hair follicles in TGF-β1 knockout mice is significantly delayed [77,78]. In the hair follicle, the Wnt signaling pathway, as a key pathway to start the hair follicle cycle, was of great significance for initiating the proliferation response of HFSCs in the bulge area; HFSCs that were treated with a Wnt pathway activator can quickly enter the proliferation period [79,80]. Moreover, Leirós et al. [81] found that Wnt pathway inhibitors (DKK-1) impair the differentiation of HFSC, and the addition of promoters (Wnt10b) can reverse this effect in AGA. 

## 6. Conclusions

This literature review showed the correlation between HL and COVID-19, analyzing the potential role of regenerative strategies in hair-loss related COVID-19. Observational studies revealed an escalated incidence of pattern HL and TE in COVID-19 patients. Psychological stress, systemic inflammation, oxidative stress, and hypoxia are potential culprits. Proinflammatory cytokines and stress hormones negatively affect the normal metabolism of proteoglycans. Reduced anagenic expression of proteoglycans is a potential mediating mechanism that connects HL to COVID-19. PRP and regenerative strategies (AD-MSCs, HFSCS, and LLLT) aim to improve scalp angiogenesis. Promoting angiogenesis and protecting the cells from ischemia are regarded as important action mechanisms in treating COVID-19-induced hair loss. The PRP technique may represent a valid regenerative strategy to improve HR-G thanks to its capacity to release several GFs, promoting the survival of dermal papilla cells during the hair cycle via the Bcl-2 protein’s activation (antiapoptotic regulator) and Akt signaling. Given the presence of only one study, further research is needed to define standardized protocols, and large-scale PRP and regenerative therapies trials based on AD-MSCs and HFSCs still need to be conducted to confirm their effectiveness.

## Figures and Tables

**Table 1:** Clinical studies on the relationship between hair loss and COVID-19.

Clinical Studies	Characteristics	Results	Year	References
Goren, A.; et al.	A descriptive study on 41 Caucasian males admitted to hospitals in Spain with a diagnosis of bilateral SARS-CoV-2 pneumonia (mean age = 58 years).	71% of the subjects were diagnosed with significant MPHL of which 39% had a severe involvement.	2020	[15]
Wambier, C.G.; et al.	Multicenter study with a follow-up on 175 patients (males and females) hospitalized with COVID-19	79% (95% CI: 70–85%) of men and 42% (95% CI: 29–55%) of women had significant PHL. These values are in sharp contrast with the expected prevalence rates among age- and race-matched populations. The prevalence of MPHL in a similar white population is expected to be at 31–53%, and that of FPHL to be at a maximum of 38%.	2020	[16]
Wambier, C.G.	Reply to “Comment on androgenetic alopecia present in the majority of patients hospitalized with COVID-19”	The data available up to date points to a considerably higher prevalence and severity of PHL among hospitalized COVID-19 patients.	2020	[18]
Wambier, C.G.; et al.	Comparing study on androgenetic alopecia in COVID-19 on age-matched epidemiologic studies and hospital outcomes with or without the Gabrin sign.	Hypoxemia leading to skin ischemia is another potential pathogenetic factor that connects lung damage secondary to SARS-CoV-2 infection with hair growth impairment.	2020	[17]
Xiong, Q.; et al.	A single-center longitudinal study on 538 COVID-19 survivors and 184 controls in Wuhan, China:	Almost half of the female participants started experiencing hair loss after being infected by SARS-CoV-2 compared to no case in the control group. 27% of affected cases experienced alopecia during their hospitalization while 73% first recognized it after being discharged. 3 to 4 months after discharge, alopecia was among the most prevalent complaints in convalescent COVID-19 patients, reported more commonly by women. Patients with higher stages of HL had worse clinical outcomes (use of ventilators and deaths). Some authors proposed the eponym ‘Gabrin sign’ to refer to the phenomenon of severe baldness in COVID-19 patients with a higher risk of unfavorable outcomes.	2020	[19]

**Table 2:** Clinical studies on the relationship between telogen effluvium and COVID-19.

Clinical Studies	Characteristics	Results	Year	References
Olds, H.; et al.	Case series of 10 patients with telogen effluvium attributed to COVID-19 infection	The mean age was 48.5 years old and 90% were female. Six of the patients were Black, one was middle eastern, and three were White. On average, the hair shedding began 50 days after the first symptom of COVID-19 infection. About 80% of these patients were treated with antibiotics, systemic corticosteroids, and/or hydroxychloroquine for their COVID-19 infection, and 70% were hospitalized. The presentations of these patients suggested that COVID-19 infection may be a significant trigger of TE.	2021	[20]
Sharquie, K.E.; et al.	Observational cross-sectional study that had been conducted during the period from September 2020 to March 2021 years on 39 patients with post COVID-19 hair loss confirmed by polymerase chain reaction (PCR) or antibody testing	39 patients were evaluated; their ages ranged from 22 to 67 years with a mean and SD of 41.3 ± 11.6 years with 36 (92.3%) females and 3 (7.69%) males. All patients with a diagnosis of acute telogen effluvium were enrolled in this study and had a laboratory-confirmed diagnosis of prior SARS-CoV-2 infection; 15 (38.46%) patients reported mild symptoms, 24 (61.53%) patients presented with moderate disease, and no patient required hospitalization. They all experienced excessive hair loss within two to three months after infection. Pull tests were strongly positive (>10–50% with a mean of 35% of pulled hair away from scalp).	2021	[21]
Moreno-Arrones, O.M. et al.	Prospective Multicentric study which enrolled 214 patients from March to August 2020 with acute telogen effluvium (ATE) that had a prior SARS-CoV-2 infection confirmed either by serological tests [e.g., detection of serum antibodies against the virus via enzyme-linked immunosorbent assays (ELISAs)] or by detection of viral RNA using real-time reverse transcription polymerase chain reaction (RT-PCR).	Mean number of days since SARS-CoV-2 diagnosis and significant hair shedding was 57.1 days (SD of 18.3). Regarding the severity of hair shedding, which was evaluated by the Sinclair Shedding Scale, 3 (4.7%) of the patients (9) had a hair shedding score of 1, 10.5% (20) of 2, 12.6 (24) of 3, 20.4% (39) of 4, 22% (42) of 5 and 29.8% (57) of 6. In 72.8% of the cases (139 patients), the ATE was active four weeks after the diagnosis. History of fever was associated (*p =* 0.04) with an increased hair shedding (Sinclair score of 5 or 6). The use of heparinoids was not associated with severity.	2021	[22]
Shome et al.	Case series study which enrolled 20 patients (all women) presenting with persistent TE starting a few weeks after recovery from Covid-19 infection, and continuing beyond six months	Management of Covid-19-induced persistent Telogen Effluvium has been unclear and futile so far. Intra-dermal administration of QR678 Neo^®^ hair growth factor formulation in the scalp reduced hair fall, improved hair regrowth, and increased hair density.	2022	[23]
Hussain et al.	A systematic review involving 465 patients diagnosed with acute TE.	The mean age was 44 years, and 67.5% were women. The most common trichoscopy findings were a decrease in hair density and empty follicles. The average duration from the onset of COVID-19 symptoms to the appearance of acute TE was 74 days, earlier than classic acute TE.	2022	[24]
Seyfi S. et al.	This observational cross-sectional study included 198 patients, confirming that TE is one of the consequences of the COVID-19 pandemic.	The study affirmed that COVID-19, via medication and stress, triggers TE.	2022	[25]
Monari et al.	A cross-sectional study which enrolled 96 patients with a diagnosis of SARS-CoV-2 pneumonia, assessed TE in 31.3% of patients, with a significant difference in sex (females 73%, males 26.7%).	The average time detected from the onset of the first symptoms to TE was 68.43 days. There were no significant associations between TE and COVID-19-related features (length of hospitalization, virologic positivity, fever’ duration), treatment characteristics, or laboratory findings. Post-infection acute TE occurs in a significant number of COVID-19 patients.	2022	[26]

## Abbreviations

HL	Hair Loss
MPHL	Male Pattern Hair Loss
FPHL	Female Pattern Hair Loss
AA	Alopecia Areata
AGA	Androgenetic Alopecia
COVID-19	Coronavirus Disease 2019
SARS-CoV-2	Severe Acute Respiratory Syndrome Coronavirus 2
ROS	Reactive Oxygen Species
IL-1	Interleukin 1
TNF-α	Tumor Necrosis Factor α
PHL	Pattern Hair Loss
IFN-γ	Interferon γ
MCP-1	Monocyte Chemoattractant Protein 1
PRP	Platelet-Rich Plasma
HFSCs	Human Follicle Stem Cells
LLLT	Low-Level Led Therapy
VEGF	Vascular Endothelial Growth Factor
GFs	Growth Factors
US	United States
FDA	Federal Drug Administration
WHO	World Health Organization
TE	Telogen Effluvium
Fibroblast	Growth Factor-7 FGF-7
MSC	Mesenchymal Stem Cells
AD-MSCs	Adipose-Derived Mesenchymal Stem Cells
HGF	Hepatocyte growth factor
DPC	Dermal Papilla Cell
ECs	Epithelial Cells
EGF	Epidermal Growth Factor (EGF)
FGF-b	fibroblast growth factor-beta
IL-6	Interleukin-6
TGF-β	transforming growth factor-beta
IGF-1	Insulin-like growth factor-1
HF	Human Follicles
BMP	Bone Morphogenetic Protein
BMPR1a	Bone Morphogenetic Protein Receptor Type 1A
M-CSF	Macrophage Colony-Stimulating
PDGF	platelet-derived growth factor
PGE2	Prostaglandin E2
PGF2α	ProstaglandinF2α
ARs	Androgen Receptors
TMPRSS2	Transmembrane Protease Serine 2
RT-PCR	Reverse transcription-polymerase chain reaction
usCRP	ultrasensitive C-reactive protein
LDH	Lactate dehydrogenase
ESR	Erythrocyte sedimentation rate
DPCs	Dermal papilla cells
